# Quality of care indicator performance was minimally changed in 2020 despite the COVID-19 pandemic

**DOI:** 10.1186/s13584-022-00516-x

**Published:** 2022-01-31

**Authors:** Alexander Konson, Michael Kuniavsky, Olga Bronshtein, Nethanel Goldschmidt, Shuli Hanhart, Hannah Mahalla, Shir Peri, Shaul Dollberg, Yaron Niv

**Affiliations:** Quality and Patient Safety Division, The National Program for Quality Indicators (NPQI), Ministry of Health, 39 Yirmeyahu St., Jerusalem, Israel

**Keywords:** Quality, Healthcare, Measurement, Indicators, Coronavirus disease 2019 (COVID-19)

## Abstract

**Background:**

In 2020, the COVID-19 pandemic affected healthcare systems throughout the world, including the management of patients and compliance rates of quality indicators.

**Objective:**

To measure the impact in Israel of the COVID-19 pandemic on the indicator-relevant caseload and compliance rates of the quality indicators reported by medical services providers within the Israeli National Program for Quality Indicators (NPQI).

**Methods:**

Data was collected from the reports made to the NPQI by participating hospitals and medical service providers. The indicator results for the number of cases and compliance rates for 2019 were compared to those from 2020. We assessed and compared the results of the quality indicators in general hospitals, geriatric hospitals and departments, psychiatric hospitals and departments, emergency medical services (EMS), and Mother and Baby health centers.

**Results:**

We found a decrease in measurable cases in 2020 relative to 2019, especially in geriatric hospitals. In most indicators, compliance rates rose in 2020. Few indicators had lower compliance rates associated with COVID-19 pandemic regulations.

**Conclusions and policy implications:**

Routine medical activity decreased in Israel in 2020 in comparison to 2019, as reflected by a decrease in cases, but compliance rates were better in most indicators. The results of our study imply that the functioning of healthcare quality measurement programs should not be interrupted during a pandemic. This not only allows measuring of the healthcare system's performance during a crisis, but also may assist in maintaining a high level of healthcare quality.

**Supplementary Information:**

The online version contains supplementary material available at 10.1186/s13584-022-00516-x.

## Background

The year 2020 was the "COVID-19 year", as the COVID-19 pandemic caused profound changes across world healthcare systems, and its effects were felt in all aspects of healthcare systems, including quality measurement. In various countries, the effects of the pandemic on the compliance rates of the quality indicators have been observed. Most reports from around the world were from an individual healthcare center or a limited number of centers, and to the best of our knowledge, studies showing the effect of the pandemic on quality indicators at the national level have not yet been published. In the USA, the Centers for Medicare & Medicaid Services (CMS) put on hold collecting and reporting to the quality indicators program and suspended specific care requirements [1, 2]. CMS indicated that data collected from the first six months of 2020 would not be used in current hospital-based performance or payment programs. This step was taken in order to improve the healthcare system's capacity to focus on preparation for a response to a potential surge of COVID-19 patients. In Israel, by contrast, there was a decision that the National Program for Quality Indicators (NPQI) activity must continue as usual. This decision stemmed from the approach that quality measurement is essential during critical situations no less than during routine healthcare. Furthermore, partial automation of the data collection and potential lack of pandemic effect on the work environments of the personnel responsible for reporting were contributing, but not the main reason for this decision. Thus, despite the difficulties involved in coping with COVID-19 pandemic challenges, the program continued to collect data and provide timely information about how the health system performed during the pandemic.

The goal of the National Program for Quality Indicators of the Israeli Ministry of Health is to promote the quality and safety of healthcare through measuring the quality of care and publishing the results to the public. Since its establishment in 2013, over 80 quality indicators in different areas of healthcare have been developed and successfully implemented at the national level in collaboration with various service providers. The program uses a fully computerized and automated process of data collection and integration followed by manual validation of a statistically significant sample of the reports; and focuses on quality indicators generated from the electronic medical record (EMR). A Business Intelligence (BI) system enables access to the data and segmentation of the results by various characteristics, for the benefit of service providers and the population.

Evaluation of the quality of healthcare provided during the pandemic is a complex process in which the assessment of changes in quality indicators plays a central and crucial role. Assessing changes in quality indicators is critical to addressing differences in the overall quality of the healthcare provided to the population and to ensure a high level of care, especially when the health system is taxed by a pandemic. Uninterrupted reporting to the program made it possible to analyze the impact of the pandemic on the quality indicators in all areas of measurement—general hospitals, geriatric hospitals and departments, psychiatric hospitals and departments, emergency medical services (EMS), and Mother and Baby health centers. We found that the number of cases relevant to most quality indicators decreased in the year 2020, as compared to 2019. Interestingly, compliance rates in the quality indicators either stayed the same or improved in 2020. Only in a small number of indicators was a decreased compliance rate observed.

## Methods

Data were collected as a part of the routine reporting to the NPQI and then examined for accuracy by independent observers before acceptance to a dedicated server (a separate, highly protected server of the Ministry of Health, dedicated to quality indicator data collection only). Senior nurses and investigators then validated a statistically significant sample of the reports that underwent statistical evaluation before the final approval. Definition of quality indicators, including denominator and numerator populations, and compliance rate calculation method, were previously described [3].

Data from all medical services providers were combined and evaluated at the national level. Newly established COVID-19 wards or those converted to COVID-19 wards from general medicine wards were excluded from quality indicators data collection. Comparison between data of 2020 and that of 2019 was performed for two parameters: the number of the relevant cases, as reflected by the size of denominator population, and the compliance rates of the indicators, as reflected by the numerator. To address the change in the indicator-relevant caseload, the denominator population of each specific indicator in 2020 was compared to the denominator population of the same indicator in 2019, and percent change was calculated. To address the change in the compliance rate of the quality indicator, the compliance rate of each specific indicator in 2020 was compared to that of the same indicator in 2019, and the difference was calculated. Institutions that did not report and indicators that were not reported for a full two years were excluded from the calculation. The actual numbers used as the denominator and numerator populations for each indicator are provided in Additional file 1: Table S1.

## Results

### General Hospitals

**Fig. 1:**
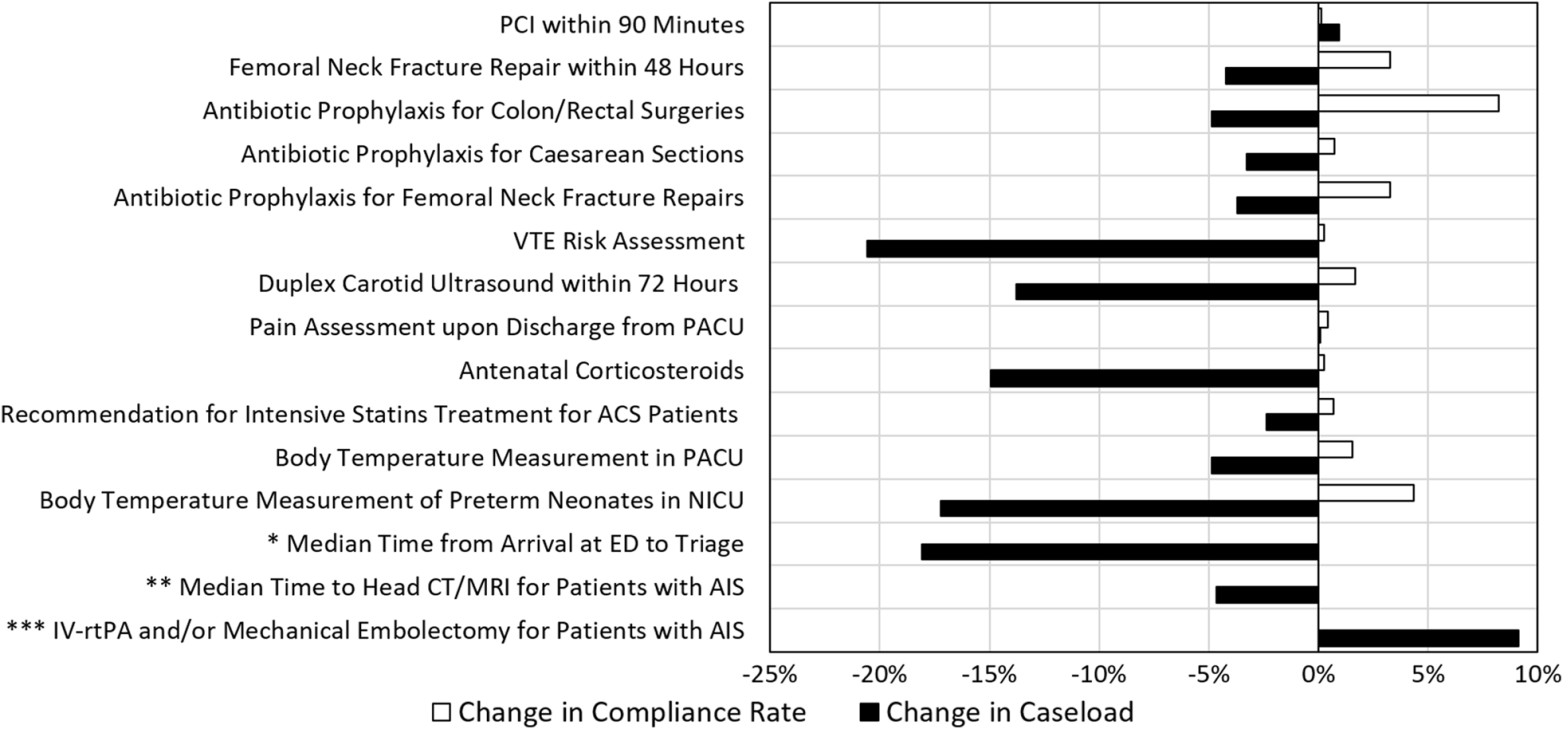
Change in the caseload and the compliance rates of the quality indicators measured in general hospitals in 2020, as compared to 2019. *Median Time from Arrival at the ED to Triage: 9 min (2019), 9 min (2020); **Median Time to Head CT/MRI for Patients with Acute Ischemic Stroke: 28 min (2019), 27 min (2020); ***IV-rtPA and/or Mechanical Embolectomy: increase of 9% in the number of treatments

In general hospitals, a clear decrease in the indicator-relevant caseload of most quality indicators was observed in 2020, as compared to 2019 (Fig. 1, black bars). Several indicators showed a more pronounced decrease in the activity than others, for example: *performance of venous thromboembolism risk assessment for patients in general medicine wards*—21% decrease, *performing a duplex carotid ultrasound within 72 h of admission to the emergency department (ED) for patients with suspected transient ischemic attack*—14% decrease, *median time from emergency room admission to clinical triage*—18% decrease. In addition, there was a decrease in the number of relevant cases of neonatology indicators: *administration of at least one course of antenatal corticosteroid in preterm deliveries*—15% decrease, the *rate of preterm neonates who had a body temperature of at least 36 °C upon arrival in the neonatal intensive care unit*—17% decrease. In the indicator *administration of intravenous thrombolytic treatment (IV-rtPA) and/or mechanical embolectomy for acute ischemic stroke*, an increase of 9% was observed in the number of treatments, possibly due to the increase of catheterization capabilities at the national level.

As shown in Fig. 1 (clear bars), although a decrease in the number of cases was observed, the compliance rates of the indicators remained stable, and some compliance rates improved. The compliance rates of the indicators *performance of PCI within 90 min for patients presenting with STEMI* and *median time from ED admission to clinical triage* remained stable. The compliance rates of the indicators *surgical repair of femoral neck fracture within 48 h* and *antibiotic prophylaxis in colon/rectal, caesarean section and femoral neck fracture repair surgeries* improved (3%, 8%, 1% and 3%, respectively). The *median time to head CT/MRI after arrival at hospital for patients with acute ischemic stroke* indicator metric decreased from 28 to 27 min.

### Geriatric Hospitals and Departments

**Fig. 2:**
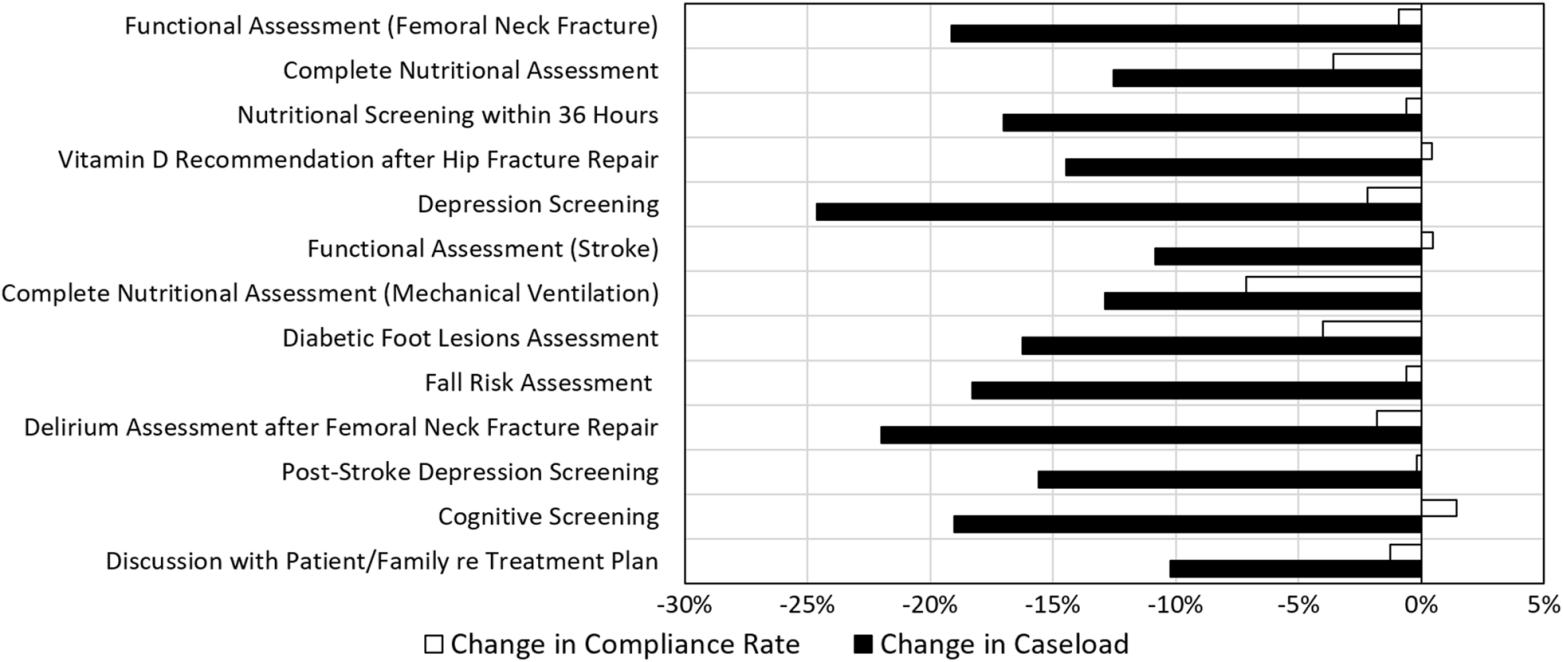
Change in the caseload and the compliance rates of the quality indicators measured in geriatric hospitals and departments in 2020, as compared to 2019

In 2020, a significant decrease of 10–25% was observed in the cases relevant to all quality indicators measured in geriatric hospitals and departments (Fig. 2, black bars). There was a significant decrease in the number of cases relevant to the admission-related indicators, including *nutritional screening*—17% decrease, *diabetic foot lesions assessment*—16% decrease and *depression screening*—25% decrease. In addition, there was a significant decrease in the number of cases relevant to post femoral neck fracture rehabilitation-related indicators, such as *functional assessment*—19% decrease, *recommendation for vitamin D administration*—14% decrease and *delirium assessment*—22% decrease.

Regarding compliance rates of the quality indicators achieved by geriatric institutions, the majority of the indicators remained unchanged or have shown only a slight decline (Fig. 2, clear bars). The compliance rates of the *recommendation for vitamin D administration after hip fracture repair*, *post-stroke depression screening* and *post-stroke functional assessment* indicators remained stable. The indicators with a slight decrease in compliance rates were: *nutritional screening* and *fall risk assessment at hospital admission*—1% decrease, *delirium assessment after femoral neck fracture repair* and *depression screening in sub-acute wards*—2% decrease, *complete nutritional assessment* and *diabetic foot lesions assessment*—4% decrease. We have also observed a 7% decrease in the compliance rate of the *complete nutritional assessment for patients on long-term mechanical ventilation* indicator, which might be due to the shortage of dieticians' workforce because of a temporary restriction for dieticians to share their work time between different institutions during the pandemic.

### Psychiatric Hospitals and Departments

**Fig. 3:**
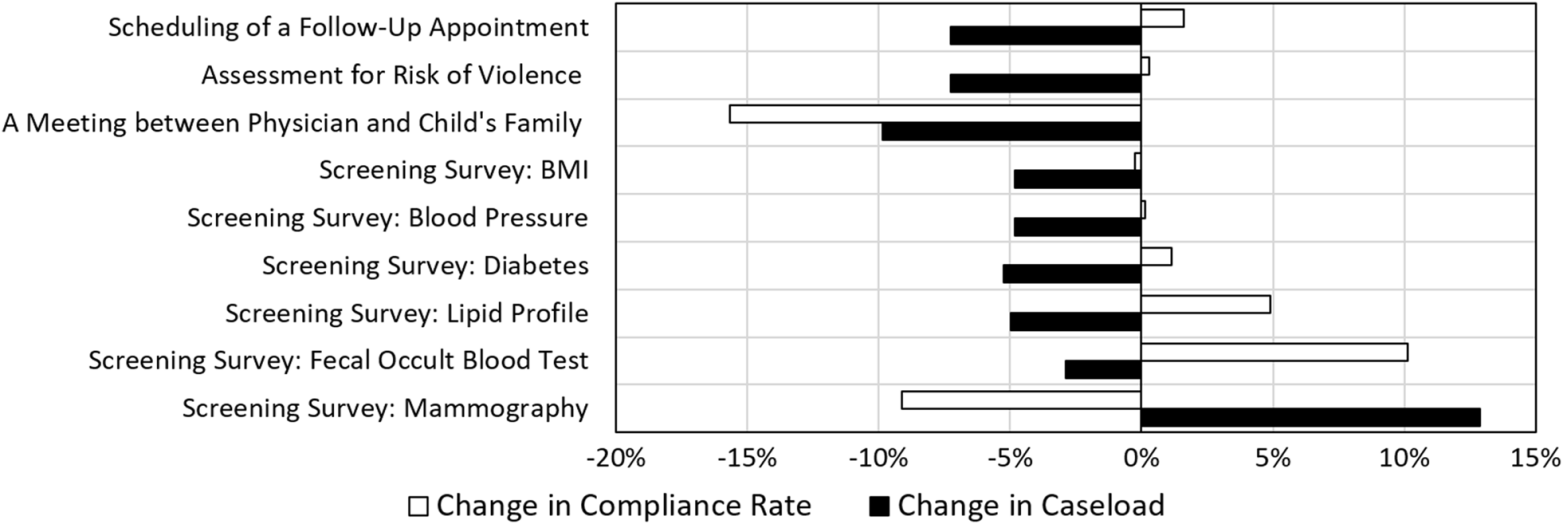
Change in the caseload and the compliance rates of the quality indicators measured in psychiatric hospitals and departments in 2020, as compared to 2019

As presented in Fig. 3 (black bars), a marked decrease in the cases relevant to the quality indicators in psychiatric hospitals and departments was observed in 2020 compared to 2019, especially in indicators related to acute hospitalization. Specifically, indicators that exhibited a prominent decrease in the number of relevant cases were *risk assessment for violence in a mental health ED*—7% decrease, *a meeting between the attending physician and the family within 5 days of the child's admission to the mental health institution*—10% decrease, and *scheduling of a follow-up community-based appointment*—7% decrease. The compliance rates of these indicators remained stable or slightly increased (Fig. 3, clear bars). In the *meeting between the attending physician and the family within 5 days of the child's admission to the mental health institution* indicator, there was a significant decrease of 16% in its compliance rate.

Other indicators in this field are prolonged hospitalization-related screening surveys: *blood pressure measurement, BMI measurement, diabetes screening, and lipid profile measurement, fecal occult blood test for colorectal cancer screening*, and *mammography*. As demonstrated in Fig. 3, these indicators showed a moderate decrease in the number of relevant cases (approximately 5%), and in most, the compliance rates increased. A substantial decrease in the compliance rate of the *mammography* screening indicator is due to the very low number of cases at the national level (Additional file 1: Table S1).

### Emergency Medical Services

**Fig. 4:**
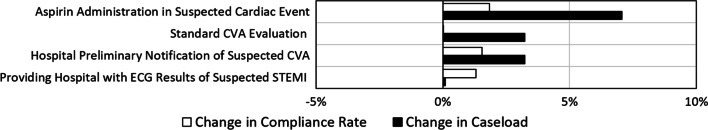
Change in the caseload and the compliance rates of the quality indicators measured in EMS in 2020, as compared to 2019

Small increases in relevant cases were observed in 2020 in the four indicators measured in the EMS area, originating from the natural growth and aging of the population (Fig. 4). Compliance rates of pre-hospital quality indicators either remained stable (*standard CVA evaluation*) or increased (*Aspirin administration in a suspected cardiac event*, *hospital notification of a suspected CVA*, and *providing the hospital with ECG results in a suspected STEMI*).

### Mother and Baby Health Centers

**Fig. 5:**
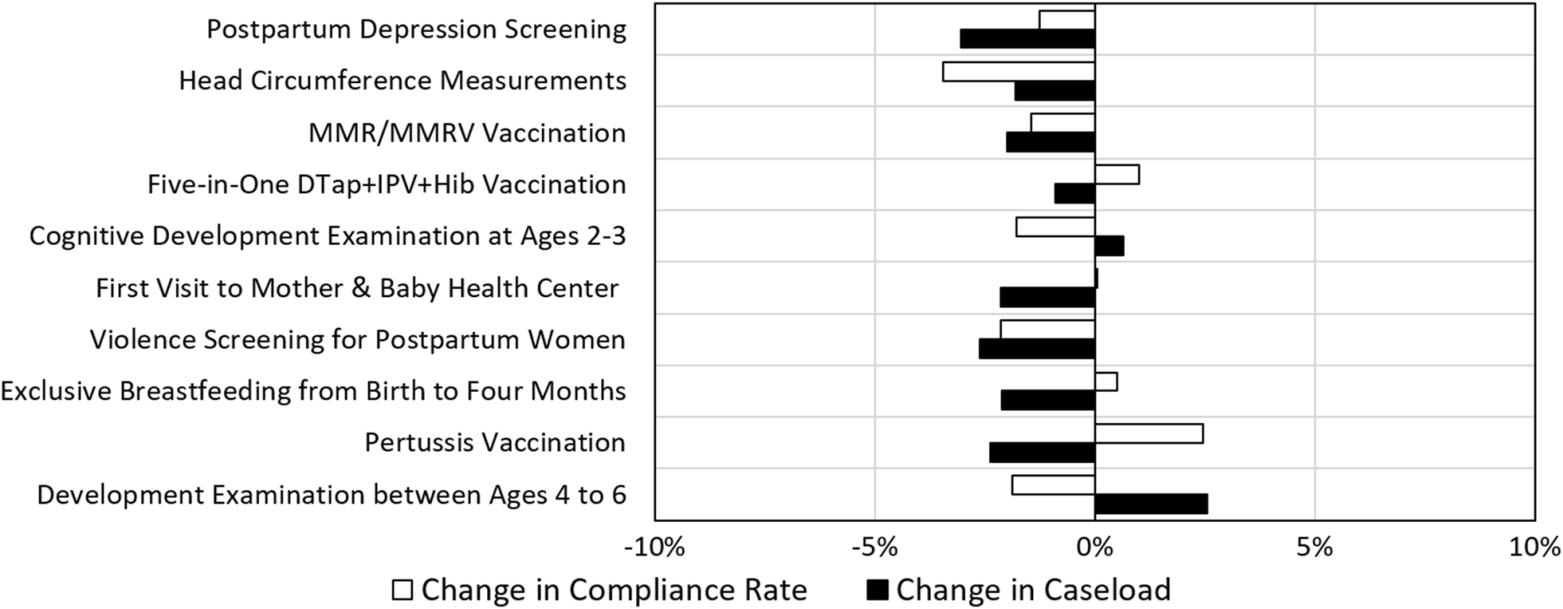
Change in the caseload and the compliance rates of the quality indicators measured in Mother & Baby health centers in 2020, as compared to 2019

In 2020, the caseload of most of the indicators measured in Mother & Baby health centers decreased moderately, by 3% at the most (Fig. 5). While there was a decrease in the number of indicator-relevant cases, compliance rates remained stable and some improved. Indicators that showed improvement in compliance rates were: *vaccination against pertussis*—2% increase, *the Five-in-One DTap* + *IPV* + *Hib vaccination*—1% increase and *maintaining exclusive breastfeeding*—1% increase. The compliance rate of *infants seen at a Mother and Baby health center within two weeks of birth ("first visit")* remained stable, and other indicators showed a very slight decrease of 1–3% in their compliance rates.

## Discussion

We present changes in the indicator-relevant caseload and the compliance rates of quality indicators enrolled in the Israeli NPQI in 2020, a year that was highly affected by the COVID-19 pandemic, as compared to 2019. The comparison was done in five types of venues—general hospitals, geriatric hospitals and departments, psychiatric hospitals and departments, emergency medical services and Mother & Baby health centers.

In general hospitals, we observed a reduction in the indicator-relevant caseload. Possible reasons for these findings include a decline in the number of referrals to the emergency departments and a consequent drop in the number of hospitalizations. These reductions were reported in several countries, including Israel ([4–8] and Additional file 2: Table S2), mainly due to the fear of infection in the hospital during the pandemic. This observation may explain the decrease in cases relevant to the indicator *median time from ED admission to clinical triage* in 2020, as compared to 2019. In addition, a decrease in the relevant caseload for indicators such as *venous thromboembolism risk assessment* and *duplex carotid ultrasound within 72 h of admission to ED for patients with suspected TIA*, may be due to the transformation of general medicine departments to COVID-19 wards. We also found a drop in the number of cases relevant to neonatology indicators of *administration of at least one course of antenatal corticosteroids* and the *rate of preterm neonates who had a body temperature of at least 36 °C upon arrival in the NICU*. The decrease in the number of premature births during the pandemic was reported elsewhere [9, 10]. The above finding also fits with a reported decrease in the number of births in Israel in 2020 ([11]; Additional file 2: Table S2). Possible causes for the decreased number of births, which are a function of the number of pregnancies in the pre-pandemic period, should be still investigated. According to our findings, despite these effects of the COVID-19 pandemic on the quality indicators-relevant caseload in general hospitals, it did not negatively affect the indicators compliance rates. One possible explanation for this finding might be the fact that general hospitals were first to join the NPQI (in 2013), and in these institutions quality measurement and improvement processes are already an integral part of the routine workflow. In addition, general hospitals (rather than geriatric or mental health institutions) possess much greater staffing depth, allowing more staff members who could be engaged to assist in both COVID-19 and routine care and thus maintain a high level of quality as well as high compliance rates with the quality indicators.

Several studies have shown the negative effect of the COVID-19 pandemic on such widely used quality indicators as *performance of PCI within 90 min for patients presenting with STEMI* and *median time to head CT/MRI after hospital arrival for patients with acute ischemic stroke* [12–14]. In Israel, however, we observed that the national compliance rate with *performance of PCI within 90 min for patients presenting with STEMI* indicator remained stable in 2020 (92%) and the *median time to head CT/MRI after arrival at hospital for patients with acute ischemic stroke* decreased from 28 min in 2019 to 27 min in 2020. These observations indicate that in-hospital quality of care for these life-threatening conditions was not affected by the pandemic.

Since the elderly population is at high risk for COVID-19, the operation of the long-term care institutions, geriatric centers, and hospitals was greatly affected by the pandemic. Geriatric institutions functioned in an unconventional environment with many challenges. This was also reflected in the predictable effect of the pandemic on quality indicators measured in these institutions. Our findings show that this area of measurement exhibited a most prominent decrease in the quality-indicators relevant caseload. During the year 2020, there was a decrease in the number of new admissions to geriatric institutions and hospital wards in Israel ([7]; Additional file 2: Table S2), due to overload and COVID-19-related regulations—such as the closing of wards or entire institutions for quarantine, and the conversion of wards into COVID-19-only wards. Accordingly, the indicators affected most, in terms of their relevance, were those measured at the admission of a new patient to the institution. Furthermore, we observed a decrease in the caseload related to rehabilitation quality indicators, probably due to a preference to perform patient rehabilitation outside of hospital settings as much as possible. Despite the many difficulties experienced by geriatric institutions with the outbreak of the COVID-19 pandemic, compliance rates of the vast majority of the quality indicators measured in geriatric institutions remained unchanged or have seen only a slight decline.

The pandemic has also challenged the mental health system, due to the uniqueness of psychiatric hospital care and the need for special arrangements to allow treatment of COVID-19 patients suffering from mental disorders requiring hospitalization. It was documented that during the pandemic, there was a considerable decrease in the number of admissions to psychiatric emergency departments [15, 16]. Correspondingly, we found that the caseload of the quality indicators measured upon the admission to psychiatric emergency departments or related to acute hospitalizations was affected more than that of other indicators in this area. Therefore, the main reason for the decrease in the caseload of these indicators is a decrease in admission rates to psychiatric emergency departments and the consequent decrease in the number of hospitalizations in mental health hospitals during the COVID-19 pandemic. Regarding the effect of the pandemic on compliance rates of the quality indicators measured in mental health wards and hospitals, a prominently affected indicator was *meeting between the attending physician and the family within 5 days of the child's admission to the mental health institution*, with a decrease of 16% in the compliance rate. This is in contrast to the other indicators since this specific indicator requires a face-to-face meeting, which was sometimes impossible due to the closure of wards to visitation or family members being in quarantine due to COVID-19 exposure. Following this finding, the NPQI approved partial use of telemedicine to enable compliance with this indicator.

We observed small increases in the number of cases relevant to the EMS quality indicators. The increase in the denominator populations of these indicators originates from the natural growth and aging of the citizens. However, based on prior NPQI experience, natural population growth is expected to provide higher increases in cases. Our observations imply that relative to the growth and aging of the population, there was a decrease in the expected number of indicator-relevant cases, which might reflect the effect of the COVID-19 pandemic on this measurement area as well.

The slight decrease in the activity of the indicators measured in Mother and Baby health centers, as compared to the other measurement areas, is due to the fact that the size of denominator population in this area depends on the number of births prior to the measurement time period, and therefore was not expected to be affected by the COVID-19 pandemic. Yet, a small decline in the caseload as well as in the compliance rates of the quality indicators in this area might be due to parents' reluctance to visit health centers during the pandemic-related national stay-at-home order periods. All indicators measured in this area require face-to-face interaction between the parent, the baby and the health center medical staff. Therefore, given the limitations during the COVID-19 pandemic, the decline in compliance rates in some indicators measured in Mother & Baby health centers was expected.

### Strengths and limitations of the study

Our study is the first to show the effect of the COVID-19 pandemic on the quality indicators-relevant caseload and compliance rates at the national level. A large set of indicators were used in different types of venues, allowing the researchers to perceive that the changes were differential depending on the type of indicator and the type of venue. A limitation of our study is that it shows the effect of the COVID-19 pandemic on the quality of routine healthcare, but no conclusions can be reached about COVID-19 care since COVID-19 wards were excluded from the study. In addition, our study does not take into consideration possible effect of the pandemic on the trend of improvement in indicators' compliance rates over prepandemic years. We aimed to show that there was a minimal change in compliance rates compared to already reached values, since significant decrease below these values may indicate a decline in the quality of routine healthcare.

## Conclusions and policy implications

In conclusion, in 2020, we observed a decrease in the caseload relevant to most indicators measured under the Israeli National Program for Quality Indicators program. However, the compliance rates did not drop, and in some indicators the rates improved. These results indicate that the quality of routine healthcare was not adversely affected by the COVID-19 outbreak in Israel. We consider this to be a great achievement of managers, quality personnel, and clinical teams who invest a great effort in providing high-quality medical services even during the challenging period of the COVID-19 pandemic. The results of our study further strengthen the approach that the functioning of the national healthcare quality measurement programs should continue during critical situations, such as the pandemic. Such an approach enables the collection of timely information about the performance of routine healthcare activities during a crisis and, more importantly, may assist in maintaining a high level of healthcare quality.

## Supplementary Information

**Additional file 1. Table S1**: Actual number of cases (denominator and numerator populations) of the quality indicators enrolled in the NPQI in 2019 and 2020.

**Additional file 2. Table S2**. Selected indicators of the healthcare utilization in Israel in 2019 and 2020.

## Data Availability

The datasets used and/or analyzed during the current study are available from the corresponding author on reasonable request.

## Abbreviations

NPQI: The National Program for Quality Indicators
COVID-19: Coronavirus disease 2019
EMR: Electronic Medical Record
BI: Business intelligence
EMS: Emergency medical services
CMS: Centers for Medicare and Medicaid Services
ED: Emergency Department
IV-rtPA: Intravenous recombinant tissue Plasminogen Activator
PCI: Percutaneous Coronary Intervention
CT: Computed tomography
MRI: Magnetic resonance imaging
ECG: Electrocardiogram
STEMI: ST Elevation Myocardial Infarction
ACS: Acute coronary syndrome
BMI: Body mass index
CVA: Cerebral vascular accident
TIA: Transient ischemic attack
AIS: Acute ischemic stroke
VTE: Venous thromboembolism
PACU: Post Anesthesia Care Unit
NICU: Neonatal Intensive Care Unit
DTap + IPV + Hib: Diphtheria and Tetanus toxoids and acellular + Inactivated Polio Vaccine + Haemophilus influenzae type b
MMR: Measles, Mumps and Rubella
