# Supplementary material for: Quality of care indicator performance was minimally changed in 2020 despite the COVID-19 pandemic
Source: Isr J Health Policy Res. 2022 Jan 31;11:9. doi: 10.1186/s13584-022-00516-x (PMC8802543; doi:10.1186/s13584-022-00516-x)
Supplement: Supplementary file 1 — Additional file 1. Table S1: Actual number of cases (denominator and numerator populations) of the quality indicators enrolled in the NPQI in 2019 and 2020. [file 13584_2022_516_MOESM1_ESM.pdf]

Additional table 1. Actual number of cases (denominator and numerator populations) of the quality indicators enrolled in the NPQI in 2019 and 2020.

Indicator	Denominator population 2019	Denominator population 2020	Numerator population 2019	Numerator population 2020
**General Hospitals**				
PCI within 90 Minutes	3,067	3,096	2,825	2,856
Femoral Neck Fracture Repair within 48 Hours	6,408	6,137	5,535	5,501
Antibiotic Prophylaxis for Colon/Rectal Surgeries	3,132	2,979	2,707	2,819
Antibiotic Prophylaxis for Caesarean Sections	28,042	27,129	26,923	26,239
Antibiotic Prophylaxis for Femoral Neck Fracture Repairs	6,276	6,043	5,693	5,679
VTE Risk Assessment	119,104	94,619	114,354	91,097
Duplex Carotid Ultrasound within 72 Hours	5,081	4,380	4,280	3,763
Pain Assessment upon Discharge from PACU	309,817	310,073	300,940	302,501
Antenatal Corticosteroids	1,808	1,538	1,786	1,523
Recommendation for Intensive Statins Treatment for ACS Patients	8,665	8,459	8,209	8,072
Body Temperature Measurement in PACU	225,275	214,350	212,112	205,117
Body Temperature Measurement of Preterm Neonates in NICU	1,498	1,240	1,289	1,121
Median Time from Arrival at ED to Triage	2,354,147	1,928,903		
Median Time to Head CT/MRI for Patients with AIS	3,641	3,471		
IV-rtPA and/or Mechanical Embolectomy for Patients with AIS	1,735	1,893		
**Geriatric Hospitals**				
Functional Assessment (Femoral Neck Fracture)	3,226	2,608	3,076	2,463
Complete Nutritional Assessment	4,808	4,206	4,232	3,551
Nutritional Screening within 36 Hours	24,380	20,228	23,927	19,727
Vitamin D Recommendation after Hip Fracture Repair	3,086	2,640	2,991	2,570
Depression Screening	2,539	1,914	2,233	1,641
Functional Assessment (Stroke)	2,267	2,021	2,162	1,937
Complete Nutritional Assessment (Mechanical Ventilation)	1,248	1,087	1,214	980
Diabetic Foot Lesions Assessment	11,594	9,709	11,069	8,882
Fall Risk Assessment	24,960	20,394	24,515	19,906
Delirium Assessment after Femoral Neck Fracture Repair	3,799	2,964	3,566	2,729
Post-Stroke Depression Screening	2,741	2,314	2,439	2,055
Cognitive Screening	12,152	9,841	10,599	8,726
Discussion with Patient/Family re Treatment Plan	3,186	2,860	2,444	2,158
**Psychiatric Hospitals**				
Scheduling of a Follow-Up Appointment	20,020	18,563	17,916	16,908
Assessment for Risk of Violence	62,780	58,210	59,623	55,450
A meeting between Physician and Child's Family	1,391	1,254	1,202	887
Screening Survey: BMI	2,073	1,973	2,027	1,924
Screening Survey: Blood Pressure	2,073	1,973	2,065	1,968
Screening Survey: Diabetes	1,753	1,661	1,509	1,449
Screening Survey: Lipid Profile	2,073	1,970	1,787	1,795
Screening Survey: Fecal Occult Blood Test	311	302	159	185
Screening Survey: Mammography	62	70	34	32
**Emergency Medical Services**				
Aspirin Administration in Suspected Cardiac Event	21,488	23,006	20,126	21,971
Standard CVA Evaluation	11,674	12,051	11,305	11,674
Hospital Preliminary Notification of Suspected CVA	11,674	12,051	10,802	11,337
Providing Hospital with ECG Results of Suspected STEMI	3,821	3,825	3,576	3,630
**Mother & Baby Health Centers**				
Postpartum Depression Screening	171,283	166,067	141,195	134,813
Head Circumference Measurements	177,609	174,385	162,834	153,858
MMR/MMRV Vaccination	179,036	175,473	130,795	125,672
Five-in-One DTap+IPV+Hib Vaccination	178,877	177,254	143,055	143,559
Cognitive Development Examination at Ages 2-3	176,611	177,736	148,824	146,608
First Visit to Mother & Baby Health Center	166,023	162,458	71,834	70,394
Violence Screening for Postpartum Women	171,673	167,181	146,932	139,528
Exclusive Breastfeeding from Birth to Four Months	82,511	80,772	56,878	56,092
Pertussis Vaccination	177,810	173,594	114,678	116,238
Development Examination between Ages 4 to 6	142,879	146,523	34,458	32,590
