# Supplementary material for: Quality of care indicator performance was minimally changed in 2020 despite the COVID-19 pandemic
Source: Isr J Health Policy Res. 2022 Jan 31;11:9. doi: 10.1186/s13584-022-00516-x (PMC8802543; doi:10.1186/s13584-022-00516-x)
Supplement: Supplementary file 2 — Additional file 2. Table S2. Selected indicators of the healthcare utilization in Israel in 2019 and 2020. [file 13584_2022_516_MOESM2_ESM.pdf]

Additional table 2. Selected indicators of the healthcare utilization in Israel in 2019 and 2020.

**Indicator**	**2019**	**2020**
Bed occupancy rate		
General care	90.7%	80.8%
Long-term care	88.0%	85.3%
Rehabilitation	101.4%	95.4%
Psychiatric care	94.7%	87.2%
Emergency department visits	3.1×10^6	2.6×10^6
Rate of ED visits per 1,000 population	341	283
Live births	182,016	177,307

Data source: the Health Information Division of the Ministry of Health (7, 8) and the Central Bureau of Statistics (11).
